# Evidence for a "Founder Effect" among HIV-infected injection drug users (IDUs) in Pakistan

**DOI:** 10.1186/1471-2334-10-7

**Published:** 2010-01-12

**Authors:** Mohammad A Rai, Vivek R Nerurkar, Suhail Khoja, Saeed Khan, Richard Yanagihara, Arish Rehman, Shahana U Kazmi, Syed H Ali

**Affiliations:** 1 Department of Biological and Biomedical Sciences, Aga Khan University, Karachi, Pakistan; 2 Retrovirology Research Laboratory, Department of Tropical Medicine, Medical Microbiology and Pharmacology, Hawaii, USA; 3 Department of Pediatrics, John A. Burns School of Medicine, University of Hawaii at Manoa, Honolulu, Hawaii 96813, USA; 4 Department of Microbiology, University of Karachi, Pakistan

## Abstract

**Background:**

We have previously reported a HIV-1 subtype A infection in a community of injection drug users (IDUs) in Karachi, Pakistan. We now show that this infection among the IDUs may have originated from a single source.

**Methods:**

Phylogenetic analysis was performed of partial gag sequences, generated using PCR, from 26 HIV-positive IDU samples.

**Results:**

Our results showed formation of a tight monophyletic group with an intra-sequence identity of < 98% indicating a "founder effect". Our data indicate that the HIV-1 epidemic in this community of IDUs may have been transmitted by an HIV positive overseas contract worker who admitted to having contact with commercial sex workers during stay abroad.

**Conclusion:**

Specific measures need to implemented to control transmission of HIV infection in Pakistan through infected migrant workers.

## Background

The first evidence of Human immunodeficiency virus (HIV) infection in Pakistan was reported in 1987 among patients receiving tainted blood or blood products [1]. Having been relatively safe from any indigenous HIV cases for around two decades, this conservative South-east Asian nation is finally registering isolated HIV outbreaks all over the country.

In 2004, in the remote desert town of Larkana, Pakistan experienced its first HIV outbreak [2]. The implicated populace was a community of Injection Drug Users (IDU), who documented an HIV prevalence approaching an outstanding 27%. Earlier, HIV prevalence in IDUs was reported by the National AIDS Control Program as bordering around 0.4% in December, 2003. After the Larkana episode, outbreaks were also recorded in other major cities of Pakistan [3]. The changing trend denotes that the country is transitioning from a low prevalence to a concentrated epidemic stage.

Apart from IDUs, other high-risk populations including truck drivers [4], commercial sex workers, including Hijras [5] have also been identified in the Pakistani context. Even though very little serological data are available, various knowledge, attitude and practice studies [3,6-9] have demonstrated an increasingly vulnerable position for a devastatingly spread of HIV in these high-risk groups.

Earlier, we have reported an HIV subtype A concentrated epidemic in a community of IDUs in Karachi, Pakistan's largest city [10]. The study presented here is an extension of that previous study on the same community of IDUs. In the previous study we showed that HIV-1 subtype A was the only subtype of HIV prevalent among this community. From the data we gathered, there were indications that this community of IDUs was infected by HIV positive overseas commercial workers (OCW) deported to Pakistan during early nineties [11]. We now present evidence that the subtype A infection in question was a single-source infection, most likely brought into the IDU community by an OCW.

## Methods

Ethical approval for this study was obtained from the Ethical Review Committee, Aga Khan University, Karachi, Pakistan. HIV-1 positive patients were selected from a community of IDUs in Karachi, the Southern Port City of Pakistan. After obtaining informed consent, 3 mL of blood was collected from 26 men (mean age, 24 years) previously diagnosed positive for HIV based on sero-testing.

DNA was extracted from the blood samples using methods previously described [10]. PCR amplification was carried out for HIV *gag *gene using two sets of primers in a nested strategy. The primers used in nested PCR were GOPF (5' CTCTCGACGCAGGACTCGGCTTGC-3', nt 683-706, HXB2) and GOPR (5'-CCAATTCCCCCTATCATTTTTGG-3', nt 2382-2404) for the first round of amplification and primers GIPF (5'-GAGGCTAGAAGGAGAGAGATGGG-3', nt 772-794) and GIPR (5'-TTATTGTGACGAGGGGTCGTTGCC-3', nt 2269-2292) for the second round of PCR.

The reaction mixture of 25 μl for both first and second round PCR contained 1× PCR buffer (5× Green GoTaq^® ^Flexi Buffer, pH 8.5), 2 mM MgCl_2_, 400 μM dNTPs and 0.3 U of *Taq *Polymerase. The first round of PCR was performed with 0.48 pmol of primers GOPF and GOPR. Thermocycle was: denaturation at 95°C for 5 min, followed by 35 cycles of denaturation at 95°C for 1 min, annealing at 58°C for 1 min and extension at 72°C for 1 min, with a final extension of at 72°C for 15 min.

1 μl of the first-round PCR product along with 0.48 pmol of the primers GIPF and GIPR was used for the second-round PCR. Thermocycle was: denaturation at 95°C for 5 min, followed by 35 cycles of denaturation at 95°C for 1 min, annealing at 60°C for 1 min and extension at 72°C for 1 min, with a final extension of at 72°C for 15 min. The amplified products were electrophoresed on 1.2% agarose gel, stained by ethidium bromide and visualized under ultraviolet light.

### Sequencing and Phylogenetic Analysis

Nested PCR products of *gag *gene were partially sequenced from Macrogen Inc, Korea, using primer GSP1 (5'-CCATCAATGAGGAAGCTGC-3', nt 1400-1418, HXB2). The nucleotide sequence spanning the p24 and p7 region of *gag *gene surrounded by the primers H1Gag1584 (5'-AAAGATGGATAATCCTGGG-3') and g17 (5'-TCCACATTTCCAACAGCCCTTTTT-3') [12], which has been considered for accurate subtyping in previous studies was analyzed. Analysis of the resulting sequences was carried out using Bioinformatic tools described previously [13]. Briefly, the sequences (comprising 460-470 bp) were compared with the sequences from the Los Alamos HIV sequence database. This was accomplished by using the *HIV BLAST *Search http://www.hiv.lanl.gov/. The samples were assigned subtypes based on the closest homology found with the subtypes in the Los Alamos database.

Using the same sequence, alignments were obtained by the *Clustal X *program (1.83) [14]. After alignment, gaps were stripped and minor manual adjustments were made using *MacClade *[15]. From these alignments, phylogenetic relationships were determined by constructing phylogenetic trees using neighbor-joining method with the help of PAUP* software [16]. Pairwise genetic distances were calculated with Kimura's two parameter method [17].

## Results and Discussion

Sequence alignment and comparison of the partial HIV-1 *gag *gene from all twenty six IDUs revealed highly congruent topologies. Based on the phylogenetic analysis, the HIV-1 strains from Pakistan clustered closely with HIV-1 subtype A sequences from Senegal and Uganda. The HIV-1 subtype A strains from Pakistan were similar to those circulating in Africa, as opposed to those in neighboring India. Moreover, the HIV-1 strains from Pakistan formed a monophyletic group validating our initial hypothesis of a possible 'founder effect' among the HIV strains from IDU in Karachi.

An identity matrix constructed by *BioEdit *software revealed more than 98% similarity among the 26 sequences analyzed, further confirming the 'founder effect' (Additional file 1). In an independent experiment, randomly selected 15 samples were analyzed in a similar way, this time using sequences from HIV *nef *gene. Once again, a phylogenetic tree was obtained showing high degree of monophylogeny among the studied samples (Additional file 2).

The HIV epidemic is still in its formative stages in Pakistan [18]. The work presented here is the first report that analyses the molecular demographics of HIV spread in this conservative South-East nation. We have demonstrated that the strains circulating amongst this select community of IDUs in Karachi are most likely a consequence of a single-source infection. This finding may well be applicable to other IDU communities in Pakistan that are recording an explosive increase in HIV infection.

**Figure 1:**
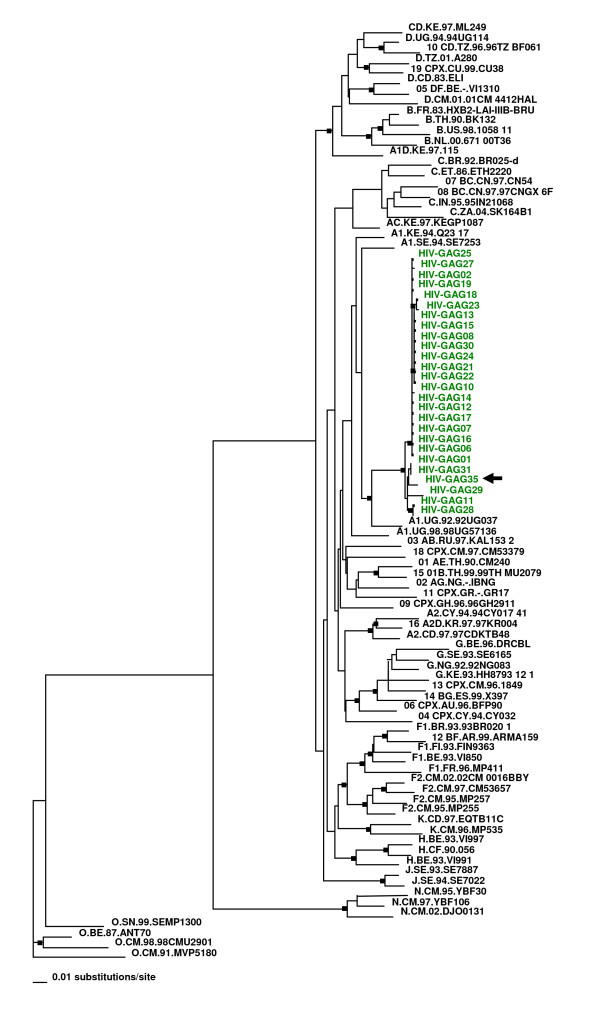
**The neighbor-joining (NJ) phylogenetic tree for the 26 sequences (shown in green) of the HIV-*gag *(p24-p7) region**. The tree was constructed by aligning the 26 sequences with HIV reference sequences from Los Alamos database. PAUP* was used to create the tree and distance matrices were generated by using Kimura two-parameter model. The sequence O.CM.91.MVP5180 was used as out group. Closed squares mark the nodes representing bootstrap values ≥ 60. The arrow on the right points to the study subject (HIV-GAG35) with a history of contact with commercial sex workers during an extended stay abroad.

In a previous report on the same community of IDUs we indicated to the possible origin of this particular endemic being the overseas commercial workers who had lived in the Middle East over extended period during which they admitted to having had sexual contact with commercial sex workers[10]. We now report deeper analysis of data from the same samples. Close phylogenetic clustering and strong identity among these HIV sequences indicates a "founder effect" of HIV in this community of IDUs, supporting the hypothesis that this entire group of 26 IDUs may have been infected with HIV from a single source. The HIV infection most likely got transmitted to the entire group through exchange of contaminated needles. The study subject HIV-GAG35 has previously reported to having had contact with commercial sex workers during a prolong stay in Sharja, Middle East [10]. During the early nineties, the subject in question was deported, after being tested positive for HIV in Sharja, to Pakistan; thereafter he developed a habit for injecting drugs. The rest of the IDUs were diagnosed positive for HIV in the year 2000 and later. Based on the fact that HIV positive HIV-GAG35 sequence clustered closely with the rest of the IDU samples (Figure 1), we speculate that this patient could be a possible source of infection in this community. That the origin of this particular infection resides in the Middle East is an attractive theory that may be corroborated further through collection and analysis of HIV positive samples from the countries in that region.

During the early nineties, an influx of HIV positive Pakistani deportees from the Middle East constituted a major threat to the transmission of infection in the country [11]. The Pakistani government did respond, albeit inadequately, to this looming disaster. Although commendable efforts were made to devise effective measures to control HIV transmission in the country, a structured program to screen and educate Pakistani migrant workers regarding sexually transmitted infections is still awaited. We have indicated before [18], and emphasize again, that with constant human traffic across borders from Middle East, Afghanistan, and India, and the prevalence of HIV in Pakistan on the rise, clear-cut policies on HIV prevention across borders is urgently warranted.

## Conclusions

The HIV/AIDS epidemic is following along the same atypical lines it has followed in the rest of Asia. Beginning from infection in the high-risk populace, the virus soon traverses the barrier to the general population. Pakistan is already experiencing multiple HIV outbreaks in the IDU community in geographically diverse regions. It would not be long before a steep rise in infection occurs, not unlike its HIV-havocked neighbor, India [19]. HIV-1 is circulating mostly within close-knit communities in Pakistan, such as the IDUs. It is therefore an early opportunity for implementing targeted HIV prevention programs in these vulnerable communities before the infection becomes an issue for the general populace.

## Competing interests

The authors declare that they have no competing interests.

## Authors' contributions

MAR: Performed PCR and sequence analysis and wrote the first draft of the paper. VRN: Developed the study and provided guidance, space and financial support for MAR's work. SKho: Performed PCR and sequence analysis for HIV *gag *gene. SKha: Helped in collection and extraction of samples. AR: Helped in accessing and surveying the IDU community. RY: carried out part of sequence analysis. SUK: Helped in analysis and interpretation of results. SHA: Supervised the study and arranged for partial funding. All authors have read and approved the submitted manuscript.

## Pre-publication history

The pre-publication history for this paper can be accessed here:

http://www.biomedcentral.com/1471-2334/10/7/prepub

## Supplementary Material

Additional file 1 **Homology among the IDU sequences**. 26 HIV *gag *sequences from IDUs were aligned using *Clustal X *and then the idenetity matrix was constructed using the *BioEdit *software. The matrix shows strong homology (98% or higher identity) among the 26 sequences compared. Click here for file

Additional file 2 **Clustering of HIV *nef *IDU sequences**. Representative phylogenetic tree, constructed by the CLUSTAL W program, using the neighbor-joining method. The tree is based on the entire *nef *gene sequence of 15 HIV-1 strains from IDU in Karachi, Pakistan (enclosed in the red box) and 66 HIV-1 sequences belonging to various HIV-1 subtypes deposited in the Los Alamos database. Click here for file
