# Supplementary figures and images for: Evidence for a "Founder Effect" among HIV-infected injection drug users (IDUs) in Pakistan
Source: BMC Infect Dis. 2010 Jan 12;10:7. doi: 10.1186/1471-2334-10-7 (PMC2820481; doi:10.1186/1471-2334-10-7)

## Slide 1
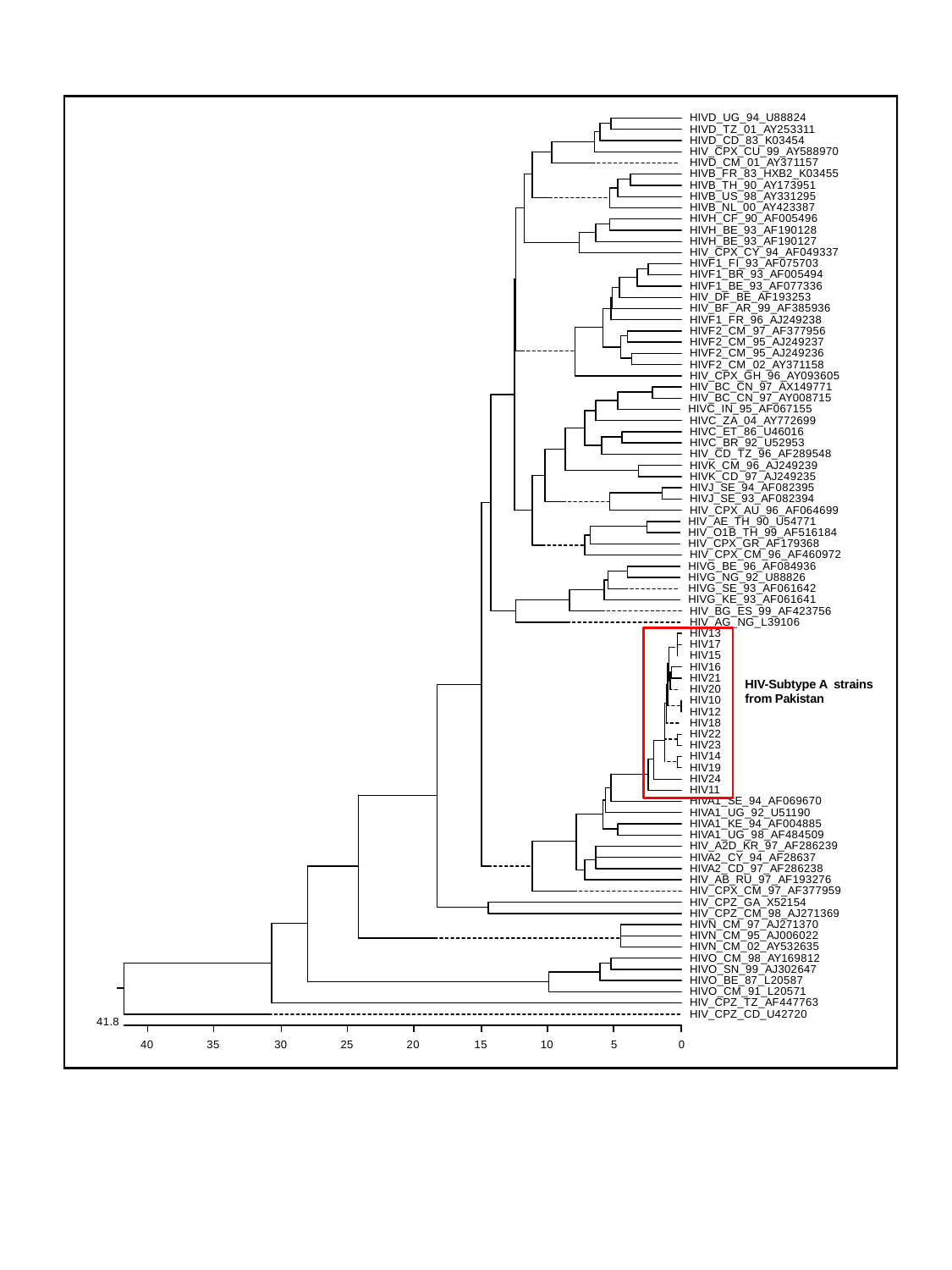

Supplement: Additional file 2 — Clustering of HIV nef IDU sequences. Representative phylogenetic tree, constructed by the CLUSTAL W program, using the neighbor-joining method. The tree is based on the entire nef gene sequence of 15 HIV-1 strains from IDU in Karachi, Pakistan (enclosed in the red box) and 66 HIV-1 sequences belonging to various HIV-1 subtypes deposited in the Los Alamos database. [file 1471-2334-10-7-S2.PPT]
